# Relations between affective music and speech: evidence from dynamics of affective piano performance and speech production

**DOI:** 10.3389/fpsyg.2015.00886

**Published:** 2015-07-08

**Authors:** Xiaoluan Liu, Yi Xu

**Affiliations:** Department of Speech, Hearing and Phonetic Sciences, University College LondonLondon, UK

**Keywords:** dynamics, emotion, piano performance, speech production, fingerings, articulatory constraints

## Abstract

This study compares affective piano performance with speech production from the perspective of dynamics: unlike previous research, this study uses finger force and articulatory effort as indexes reflecting the dynamics of affective piano performance and speech production respectively. Moreover, for the first time physical constraints such as piano fingerings and speech articulatory constraints are included due to their potential contribution to different patterns of dynamics. A piano performance experiment and speech production experiment were conducted in four emotions: anger, fear, happiness and sadness. The results show that in both piano performance and speech production, anger and happiness generally have high dynamics while sadness has the lowest dynamics. Fingerings interact with fear in the piano experiment and articulatory constraints interact with anger in the speech experiment, i.e., large physical constraints produce significantly higher dynamics than small physical constraints in piano performance under the condition of fear and in speech production under the condition of anger. Using production experiments, this study firstly supports previous perception studies on relations between affective music and speech. Moreover, this is the first study to show quantitative evidence for the importance of considering motor aspects such as dynamics in comparing music performance and speech production in which motor mechanisms play a crucial role.

## Introduction

### Background

Music and speech reflect fundamental aspects of human capacities (Juslin and Laukka, 2003; Patel, 2008). The parallels between music and speech have been attracting scholarly interest for a long period (Fonagy and Magdics, 1963; Sundberg, 1982; Scherer, 1995), with attempts to compare the two from a wide range of perspectives: prosody (Scherer, 1995), semantics (Seifert et al., 2013), syntax (Lerdahl, 2013), evolution (Cross et al., 2013), neurocognitive mechanisms (Steinbeis and Koelsch, 2008), and facial expressions (Carlo and Guaitella, 2004; Livingstone et al., in press). Particularly, an increasing amount of attention has been given to using perceptual tests for acoustic comparisons between affective music and speech, as they are two important means of emotion communication (Buck, 1984; Wilson, 1994; Juslin and Laukka, 2003) which is crucial for maintaining social bonds in human society (Ekman, 1992). The majority of comparative studies show that perceptually, acoustic cues (pitch, intensity, and duration) of affective music and speech are similar (Juslin and Laukka, 2003; Curtis and Bharucha, 2010; Ilie and Thompson, 2011).

Admittedly, perception tests have brought valuable insight into the acoustic relations between affective music and speech, but sometimes individual variation can significantly influence perceptual judgments which could lead to unreliable or even contradictory results (cf. Juslin and Sloboda, 2013). Therefore, this study aims to compare affective music and speech from a different perspective by using affective piano performance and speech production with a special focus on dynamics. This is because compared with the vast amount of perception studies, research using production experiments on affective music performance is on the rise only in the past 20 years, thanks to the advent of music technology that makes quantifying music performance easier than before. Particular interest has been given to piano/keyboard performance due to the availability of MIDI, 3D motion capture cameras, digital acoustic pianos (Palmer, 2006). However, to our knowledge, strictly controlled experiments that directly compare affective piano performance with speech production are rare. In this study we focus on dynamics in the comparison between the two domains with the inclusion of fingerings in piano performance and articulatory constraints in speech production. The reasons will be elaborated on in the following sections.

### Dynamics of piano performance

In studies of affective piano performance, dynamics have received less attention than timing, although they are equally important (Repp, 1996; Gabrielsson, 2003). The reason is that unlike timing which can be easily measured by metronome and hence has been systematically examined in a scientific way for over a decade (Repp, 1992a,b, 1994a,b, 1995, among others), dynamics are more difficult to measure. This could be partly due to perceptual difficulty in precisely distinguishing different levels of dynamics (e.g., *forte* and *mezzoforte*) or technical challenges in filtering out unwanted acoustic artifacts (Repp, 1996).

Therefore in this study we decide to examine piano dynamics from a different perspective, i.e., at the kinematic level of dynamics which reflects “the varying forces of the pianist's finger movements on the keyboard” (Repp, 1996, p. 642) by using a modified Moog PianoBar scanner (cf. McPherson, 2013). It is a portable scanner that can be rapidly attached to any acoustic piano keyboards. Using an optical reflectance sensing mechanism, the modified PianoBar scanner continuously detects key movements. Quantitatively, the scanner returns the values of continuous key positions (key displacement) and the time taken for fingers to reach each key position during one keystroke. As a result, multiple different dimensions of each key press, velocity and peak velocity (i.e., the maximum value in a continuous velocity trajectory) of key movement during each keystroke can be extracted from continuous key position data, following a similar approach to McPherson and Kim (2011). The multidimensions of key touch quantitatively returned by the scanner can provide an ideal platform for examining the interaction between pianists' expressive intention and their piano key touch (cf. McPherson and Kim, 2013).

Literature on mechanics of skilled motor movement (such as speech production and music performance) suggests that dynamics of motor movement are related not only to peak velocity but also to the movement amplitude, i.e., the peak velocity should be divided by the movement amplitude in order to compare dynamics of movement of different sizes (Nelson, 1983; Ostry et al., 1983; Ostry and Munhall, 1985). Therefore, in the context of piano performance, since each keystroke may correspond to different degrees of key displacement (i.e., different amplitudes of key movement), it is necessary to factor in key displacement at the point of peak velocity to yield the kinematic dynamics of each keystroke which reflects pianists' finger force (Minetti et al., 2007). Similar approach can also be found in Kinoshita et al. (2007) where key displacement was taken as a factor in comparing finger force under the conditions of different types of key touch.

The examination of kinematic dynamics needs to take into account the role of fingerings. This is because in piano performance, alternative fingerings can be used for the same piece of music, which is unlike playing other instruments. Usually, different fingering strategies can reflect how pianists intend to interpret the structure, meaning and emotion of music in which dynamics play an important role (Neuhaus, 1973; Bamberger, 1976; Clarke et al., 1997). Parncutt et al. (1997) established a set of hypothetical rules of right-hand fingerings according to ergonomic difficulty such as the extent of hand spans, the involvement of weak fingers, and the coordinated playing on black and white keys. Of particular importance are hand spans and weak fingers. This is because the extent of hand spans can affect the degree of tension and physical effort of fingers (Parncutt et al., 1997). Weak fingers usually refer to the fourth and fifth fingers (Parncutt et al., 1997) which can constrain the flexibility of finger movement because of the hand's anatomical structure: unlike the thumb and index fingers which are relatively independent, the middle, ring and little finger are closely linked to each other via the flexor digitorum profundus (FDP) tendons because they share a common muscle belly (Gunter, 1960). Moreover, the flexor digitorum superficialis (FDS) is especially responsible for the coupling between the fourth and fifth fingers (Baker et al., 1981; Austin et al., 1989). Nevertheless, whether weak fingers can significantly influence piano performance is still a matter of debate. As pointed out in Kochevitsky (1967), Neuhaus (1973), and Sandor (1981), weak fingers are not necessarily weak; instead, they are often strong enough to meet the demand of different levels of playing, especially octave playing.

### Dynamics of speech production

With regard to speech, articulatory effort which reflects “force of articulation” (Malécot, 1955) is the counterpart of finger force in piano performance. Articulatory effort is essentially a neuromuscular phenomenon. Electrochemical reaction of nerve impulses triggers the activation of articulator muscles (Kirchner, 1998). Full contraction of articulator muscles occurs when agonist muscle activity outweighs the antagonist muscle activity under the condition of repeated neuron firing (Clark and Yallop, 1990). Articulatory effort is therefore the sum action of the neuron firing of each articulator muscle (Kirchner, 1998). However, direct measurements of the neuron firing of each articulator muscle are clearly too intrusive and complex to perform. Therefore, indirect measurements have been put forth through examining phenomena related to articulatory gestures: articulator displacement (Lindblom, 1983), clear speech (Uchanski, 2008), fricative closures (Lavoie, 2001), trill articulation (Padgett, 2009), assimilation (Lindblom, 1983), all of which require great articulatory effort. Correspondingly, speech production models as in Lindblom and Sundberg (1971), Westbury and Keating (1986), Kirchner (1998), have been established in an attempt to quantify articulatory effort. However, the aforementioned measurements of articulatory gestures run the risk of not capturing articulatory phenomena large enough for statistically significant differences (Kaplan, 2010); in addition, the proposed models would oversimplify the reality of speech articulation which often involves much finer details than what the models can accommodate (Kaplan, 2010).

Hence, different alternatives are worth exploring. One such example is to use formant dynamics (i.e., trajectories and velocity) as an indicator of articulatory effort (cf. Cheng and Xu, 2013). Admittedly, one could argue formant dynamics may not be a reliable indicator of articulatory effort given the fact that there does not exist a one-to-one mapping between acoustics and articulation. Nevertheless, direct measurements on articulators as has been pointed out above do not capture the whole picture of articulatory movement either (cf. Cheng and Xu, 2013 for more examples and discussions). Acoustic signals, on the other hand, have been argued to provide reliable information for phonetic characteristics of segments and suprasegments with theoretical (Lindblom, 1990) and experimental evidence (Perkell et al., 2002). In addition, acoustic and articulatory measurements can produce similar dynamic patterns: the evidence is that the linear relations between F0/formant velocity and F0/formant movement amplitude (Xu and Wang, 2009; Cheng and Xu, 2013) in acoustics are similar to those in articulation (Kelso et al., 1985). Therefore, it is justifiable to use acoustic characteristics of formant dynamics to analyze articulatory effort.

In speech, formant patterns tend to be affected by articulatory constraints (e.g., articulatory pressure and distance) in different suprasegmental and segmental contexts (Erickson et al., 2004; Kong and Zeng, 2006). Tone languages such as Mandarin can be a typical platform for investigating articulatory pressure in different suprasegmental contexts: In Mandarin, there are five types of tones—High (tone 1), Rising (tone 2), Low (tone 3), Falling (tone 4), and Neutral (tone 5). Tone 2 + tone 2 and tone 4 + tone 4 create high articulatory pressure while tone 3 + tone 1 create weak articulatory pressure. The reason is that as reported in Xu and Wang (2009), successive rising tones (i.e., tone 2 + tone 2) or falling tones (tone 4 + tone 4) create much larger articulatory pressure than other tonal combinations because each involves two movements within one syllable. Successive static tones (tone 3 and tone 1), in contrast, have much smaller articulatory pressure because only a single movement is needed within each syllable. With regard to the segmental dimension, diphthongs (i.e., two adjacent vowels) can be used because they are categorized into wide and narrow diphthongs according to their articulatory distance: wide diphthongs (e.g., /ai/, /au/, /ɔi/) have wider articulatory distance between the initial and final vowel and hence involve greater articulatory movement of speech organs. Narrow diphthongs (e.g., /ei/, /əu/) have narrower articulatory distance between the initial and final vowel and hence the articulatory movement is not as large as wide diphthongs.

### Motivations for this study

Theoretically, motion for a long time has been an important platform for investigating music, i.e., how physical motion is associated with sound patterns subsequently generated (Sundberg, 2000). Human voice is a direct reflection of such motion-to-sound mapping through physical coordination of articulatory gestures; meanwhile, performance of musical instruments is another way of mapping motion to sound through the use of tonguing, breathing, and fingering (Palmer et al., 2007, 2009). Therefore, similar to speech production, music performance can be conceptualized as a “sequence of articulatory movements resulting in a continuous acoustic wave” (Palmer et al., 2007, p. 119). In the context of piano performance, fingers can thus be perceived as “articulators” for pianists to articulate their interpretation of music. Indeed, experimental results on piano performance (Winges et al., 2013) show that speech production phenomenon such as coarticulation also exists in pianists' finger movement during performance. This is not surprising given the fact that both speech production and piano performance are under neuromuscular control (Winges et al., 2013) and essentially both domains require skilled motor movements following similar physical mechanisms of dynamics (Grillner et al., 1982; Nelson, 1983; Ostry et al., 1983; Winges et al., 2013; van Vugt et al., 2014). In the context of motor movement, force is a crucial component contributing to the dynamics of physical movements (Stein, 1982). Therefore, it is reasonable to compare articulatory effort with force of other types of motor movements such as finger force (Gentil and Tournier, 1998; Ito et al., 2004; Loucks et al., 2010). As discussed in previous sections, the kinematic dynamics of keystroke reflect pianists' finger force and the formant dynamics of speech reflect speakers' articulatory effort. Since music performance and speech are two important platforms for humans to communicate emotion (Juslin and Laukka, 2003), plus the fact that these two domains are essentially skilled motor movements following similar physical mechanisms of dynamics as discussed above, it is therefore justifiable to compare music performance and speech production in the context of emotion using dynamics of motion (i.e., kinematic dynamics of keystroke and formant dynamics of speech production) as a measurement parameter. To our knowledge, such comparison is currently missing in literature and we believe it is worth bridging the gap.

In addition, one may wonder how piano fingerings (Section Dynamics of Piano Performance) and articulatory constraints (Section Dynamics of Speech Production) can relate to each other. Anatomically, articulation refers to motor movement caused by skeletal muscle contraction (Tortora, 2002). Hence typical human motor movements such as speech production or music performance are effectively muscular articulation. There is no wonder, therefore, that pianists' fingers are always referred to as “articulators” expressing pianists' interpretation of music. Different fingerings involve different degrees of hand span and alternation between strong and weak fingers, which consequently lead to different degrees of finger muscular tension (Parncutt et al., 1997). Similarly in speech, different degrees of articulatory constraints are involved as discussed in Section Dynamics of Speech Production. Both finger muscular tension and speech articulatory pressure can be considered as physical constraints on motor movements such as piano performance and speech production (Nelson, 1983; Winges et al., 2013). Therefore it is the physical constraints triggered by different fingerings or articulatory constraints that relate the two domains to each other. Despite the importance of fingerings and articulatory constraints reviewed above, it is still unknown whether they interact with emotion in piano performance and speech production. This serves as another motivation for this study.

Four of the basic emotions (Ekman, 1992) are chosen: anger, fear, happiness, and sadness. One may wonder why a discrete model of emotion (Ekman, 1992; Panksepp, 1998) has been chosen rather than a dimensional approach such as Russell's circumplex model (1980). This is because firstly, so far no theoretical consensus has been reached as to which approach is better than the other for modeling emotion (for a recent summary of theoretical debates, see Zachar and Ellis, 2012). More importantly, the two approaches are not necessarily in conflict with each other as recent affective neuroscience studies (e.g., Panksepp and Watt, 2011) have suggested that the differences between the two may well be insignificant given the fact that both approaches share many common grounds in explaining cognitive functions of emotion. Since it is not the purpose of this study to test which model is better, a discrete model of affect is adopted. Among the “big six” emotions (Ekman, 1992), vocal disgust usually cannot be elicited satisfactorily under laboratory conditions (cf. Scherer, 2003); musical surprises can be very complicated often requiring sharp contrast in compositional structure (Huron, 2006) which is out of the scope of this study. Hence, only the remaining four of the “big six” emotions are chosen. The research questions to be addressed are.

Are dynamics of piano performance (i.e., finger force) similar to or different from dynamics of speech production (i.e., articulatory effort) under the condition of the four emotions? Do fingerings and articulatory constraints interact with emotion in their influence on the dynamics of piano performance and speech production respectively?

## Experiments

### Experiment 1: The piano experiment

#### Stimuli

**Figure 1:**
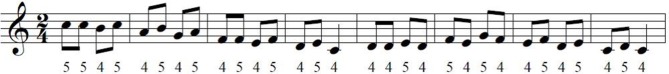
**The small-weak condition (SW): small hand span (i.e., fingers are at their natural resting positions) with only weak fingers, i.e., the ring (4) and little (5) fingers involved**.

**Figure 2:**
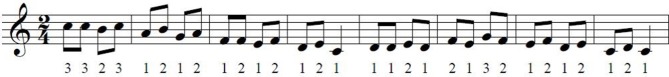
**The small-strong condition (SS): small hand span (i.e., fingers are at their natural resting positions) with only strong fingers, i.e., the thumb (1), index (2) and middle (3) fingers involved**.

**Figure 3:**
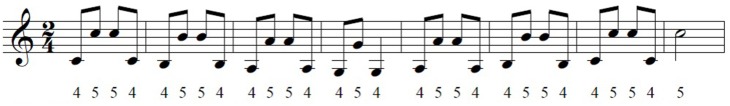
**The large-weak condition (LW): large hand span (i.e., fingers stretching across an octave) with only weak fingers, i.e., the ring (4) and little (5) fingers involved**.

**Figure 4:**
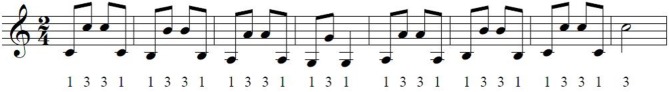
**The large-strong condition (LS): large hand span (i.e., fingers stretching across an octave) with only strong fingers, i.e., the thumb (1) and middle (3) fingers involved**.

Two excerpts of music were composed for this study. According to the above reviews on fingerings, hand span and weak fingers should be the primary focus. Therefore, the two excerpts were composed corresponding to small and large hand span, respectively. Small hand span is where fingers are at their natural resting positions on the keyboard, i.e., without needing to extend far beyond the resting positions to reach the notes (Sandor, 1981). Large hand span is where fingers need to extend far beyond their resting positions, which usually involves stretching at least an octave (Parncutt et al., 1997). Meanwhile, each excerpt was to be played with strong finger combinations (the thumb, index, and middle fingers) and weak finger combinations (the ring and little fingers). In addition, given the fact that right and left hands tend to have different patterns in piano performance (Minetti et al., 2007), only the right hand is involved in this experiment to avoid theoretical and practical complexities. Hence altogether there are four levels of fingerings for this study: small-weak (SW), small-strong (SS), large-weak (LW), large-strong (LS). To avoid confounding effects, all excerpts have musically neutral structure, i.e., without having overtly emotional implications. Figures 1–4 demonstrate the fingering design.

#### Participants and procedure

This experiment was approved by the Committee on Research Ethics at University College London. Eight professional pianists (four females, Mean = 26 years, *SD* = 2.2, all right-handed) from London were recruited to play the excerpts according to the fingerings provided on scores. They have been receiving professional piano training for an average of 20 years. They were instructed to play each of the excerpts with four emotions: anger, happiness, fear, and sadness. Each excerpt per emotion was repeatedly played three times in a quiet room. Admittedly, lacking ecological validity can be a problem with this method, i.e., it deviates from the reality of music making in that firstly, performances usually take place in concert halls; secondly, different emotions are often expressed by different pieces of music (cf. Juslin, 2001 for references therein). Nevertheless, real music making settings often cannot be scientifically controlled, i.e., it is impossible to filter out confounding factors coming from the acoustics of concert halls and audience. Moreover, it is hard to judge whether it is the way music is performed or the melody of music that leads the listeners to decide on the emotional categories if different pieces of music are used for different emotions (Juslin, 2000, 2001). Therefore, conducting the experiment in a scientifically controlled way is still the better option if validity of the results is the priority.

As introduced in Section Dynamics of Piano Performance, a Moog PianoBar scanner was attached to the keyboard of a Bösendorfer grand piano. Finger force is reflected by keystroke dynamics which were calculated according to the formula: $dynamics=\frac{peak\;velocity\;of\;each\;keystroke\;(Vp)}{maximum\;piano\;key\;displacement\;(d)}$ (Vp/d henceforth) because of the need to consider movement amplitude (i.e., displacement) in relation to peak velocity to reflect kinematic dynamics as reviewed above. More specifically, $Vp=\frac{maximum\;piano\;key\;displacement\;(d)}{time\;taken\;to\;reach\;the\;maximum\;displacement\;(t)}$, and so the ratio $Vp/d=\frac{d}{t}\times \frac{1}{d}=\frac{1}{t}$. The unit of displacement is mm and that of time is sec. The data were obtained by an external computer attached to one end of the PianoBar. A Matlab script was written for computing dynamics according to the formula.

There were altogether 8 (pianists) ^*^ 4 (emotions) ^*^ 4 (fingerings) ^*^ 3 (repetitions) = 384 episodes performed by the pianists. A follow-up perceptual validation test was carried out: sixteen professional musicians (10 females, Mean = 28 years, *SD* = 1.5) were asked to rate each emotion ^*^ fingering episode on a 1–5 scale. 1 represents not at all angry/fearful/happy/sad while 5 represents very angry/fearful/happy/sad. The top 8 ranked episodes (out of 24) for each emotion ^*^ fingering were selected. The mean score for each emotion ^*^ fingering was 4.03.

#### Results of the piano experiment

A Two-Way repeated measures ANOVA was performed to examine the effect of emotion (four levels: anger, fear, happiness, and sadness) and fingerings (four levels: small-weak, small-strong, large-weak, large-strong). The results (Table 1) demonstrate that both factors play significant roles in finger force reflected by Vp/d. The interaction between the two factors is also significant.

**Table 1:** **Mean Vp/d of the four levels of emotion (A, anger; F, fear; H, happiness; S, sadness) and the four levels of fingerings (SW, small-weak; SS, small-strong; LW, large-weak; LS, large-strong)**.

	**Anger**	**Fear**	**Happiness**	**Sadness**
Mean Vp/d	25.2	17.3	22.9	5.8
Standard deviation	1.8	4.9	2.1	1.2
	**SW**	**SS**	**LW**	**LS**
Mean Vp/d	13.7	17.8	19.4	20.5
Standard deviation	2	1.8	2.6	1.5

The means of keystroke dynamics (Vp/d) for each condition are displayed in Table 2 and *Post-hoc* Tukey HSD tests (Table 3) reveal more detailed patterns: anger and happiness have significantly higher dynamics than fear and sadness. The differences between anger and happiness are non-significant. Fear has significantly lower dynamics than anger and happiness but it is still significantly higher in dynamics than sadness. With regard to the factor of fingerings, the Tukey tests demonstrate that weak fingers in large hand span (the LW condition) do not produce significantly different dynamics from strong fingers in large hand span (the LS condition). However, under the condition of small hand span, weak fingers tend to produce significantly lower dynamics than strong fingers.

**Table 2:** **Results of the Two-Way repeated-measures ANOVA of emotion and fingerings on keystroke dynamics (as reflected by Vp/d)**.

	***F***	***df***	***p***	***η*^2^_*p*_**
Emotion	8.26	3.21	<0.001	0.31
Fingerings	4.05	3.21	<0.05	0.16
Emotion ^*^ fingerings	2.17	9.63	<0.05	0.13

**Table 3:** **Results of *Post-hoc* Tukey tests on means of the four levels of emotion (A, anger; F, fear; H, happiness; S, sadness) and the four levels of fingerings (SW, small-weak; SS, small-strong; LW, large-weak; LS, large-strong)**.

*p*	A vs. F	A vs. H	A vs. S	F vs. H	F vs. S	H vs. S
	<0.05	>0.05	<0.001	<0.05	<0.05	<0.001
*p*	SW vs. LS	SW vs. LW	SW vs. SS	LS vs. LW	LS vs. SS	LW vs. SS
	<0.01	<0.01	<0.05	>0.05	>0.05	>0.05

**Figure 5:**
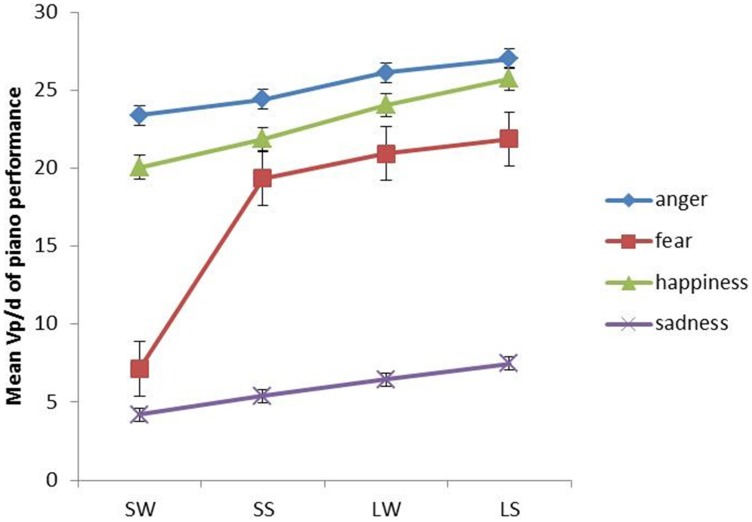
**The interaction between emotions and fingerings in terms ofVp/d in piano performance**. Error bars represent the standard error of the mean.

In terms of the interaction between emotion and fingerings, Figure 5 shows that the most obvious interaction is between fear, large-strong (LS), large-weak (LW), and small-strong (SS) fingering conditions. For all of the aforementioned fingerings, fear has significantly higher (*p* < 0.05) dynamics than sadness according to a series of *post-hoc* Tukey tests, although it is still significantly lower (*p* < 0.05) than anger and happiness. Between the LS, LW, and SS conditions in fear, the differences are non-significant. For anger, happiness and sadness, large hand span generates higher dynamics than small hand span, but the differences are non-significant, i.e., regardless of whether the hand span is large or small, the dynamics are on average always high for anger and happiness while for sadness they are always low. Therefore, the contrast in dynamics between different fingerings is evident under the condition of fear only.

### Experiment 2: The speech experiment

#### Stimuli

The stimuli consist of six sentences divided into two sets (Tables 4, 5), with tones and vowels being the two variables. The purpose is to use the two variables to test the respective impact of suprasegmental and segmental constraints on formant dynamics. According to the reviews in Section Dynamics of Speech Production, tone 2 + tone 2 and tone 4 + tone 4 are used to create high articulatory pressure. Tone 3 + tone 1 is used to create low articulatory pressure. Meanwhile, a wide diphthong /au/ is used for long segmental distance and a narrow diphthong /əu/ is used for short segmental distance. *Cuilaoyao* and *Cuilouyou* are compound words denoting a person's name.

**Table 4:** **The first set of the stimuli in which the numbers of the syllables represent the five lexical tones in Mandarin: 1 for H (High tone), 2 for R (Rising tone), 3 for L (Low tone), 4 for F (Falling tone), and 5 for N (Neutral tone)**.

	*lao2[lau]* work	*yao2[jau]* distant				
*cui1* surname	*lao3[lau]* old	*yao1[jau]* waist	*nian4* read	*shu1* book	*qu4* aspect	*le5* particle
	*lao4[lau]* flood	*yao4[jau]* medicine				

*IPA transcriptions for the key words laoyao are provided in brackets. Translation: cuilaoyao has gone to read a book*.

**Table 5:** **The second set of the stimuli in which the numbers of the syllables represent the five lexical tones in Mandarin: 1 for H (High tone), 2 for R (Rising tone), 3 for L (Low tone), 4 for F (Falling tone), and 5 for N (Neutral tone)**.

	*lou2[ləu]* building	*you2 [jəu]* oil				
*cui1* surname	*lou3[ləu]* hug	*you1[jəu]* good	*nian4* read	*shu1* book	*qu4* aspect	*le5* particle
	*lou4[ləu]* drip	*you4[jəu]* right				

*IPA transcriptions for the key words louyou are provided in brackets. Translation: cuilouyou has gone to read a book*.

#### Measurement of formant dynamics

As reviewed in Section Dynamics of Speech Production, formant dynamics are an important factor reflecting the articulatory effort of speech production. Formant peak velocity, i.e., “the highest absolute value in the continuous velocity profile of the (formant) movement” (Cheng and Xu, 2013, p. 4488), and the displacement/amplitude of the formant movements are particularly related to articulatory effort [cf. Cheng and Xu (in press) for further discussion]. The peak velocity is measured in the following way (Xu and Wang, 2009, p. 506):

“Positive and negative extrema in the velocity curve correspond to the rising and falling ramps of each unidirectional pitch (formant) movement. A velocity curve was computed by taking the first derivative of an F0 (formant) curve after it has been smoothed by low-pass filtering it at 20 Hz with the Smooth command in Praat. Following Hertrich and Ackermann (1997), the velocity curve itself was not smoothed so as not to reduce the magnitude of peak velocity.”

**Figure 6:**
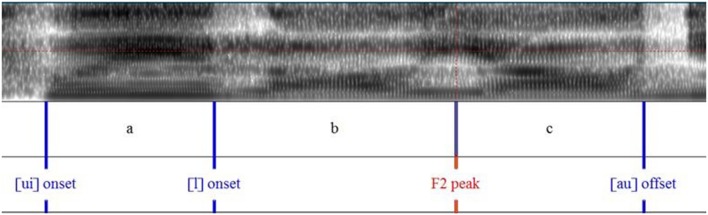
**Syllable segmentation and labeling of the sentence “*Cui laoyao nian shu qu le*”**.

**Figure 7:**
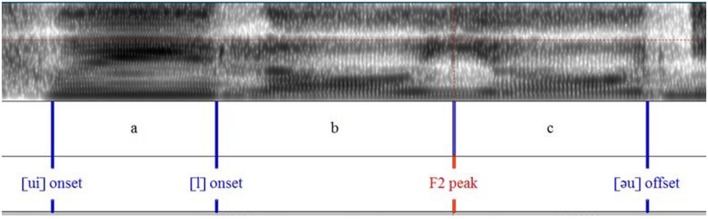
**Syllable segmentation and labeling of the sentence “*Cui louyou nian shu qu le*”**.

Figures 6, 7 show the measurement points taken from F1 and F2 formant contours. This allows the calculation of the ratio of formant peak velocity (Vp) to maximum formant displacement (d), henceforth Vp/d. It reflects articulatory effort/vocal vigorousness (Cheng and Xu, 2013, in press). Similar to the piano experiment, $Vp=\frac{maximum\;formant\;displacement\;(d)}{time\;taken\;to\;reach\;the\;maximum\;displacement\;(t)}$, and so the ratio $Vp/d=\frac{d}{t}\times \frac{1}{d}=\;\frac{1}{t}$. The unit of displacement is Hz and the unit of time is sec.

Table 6 lists the values extracted from the measurement points for the calculation of Vp/d for F1 and F2.

**Table 6:** **Values taken from the measurement points a, b, c for the calculation of Vp/d**.

minF1a: F1 minimum in *a*	minF2a: F2 minimum in *b*
maxF1a: F1 maximum in *b*	maxF2a: F2 maximum in *a*
minF1b: F1 minimum in *b-c*	minF2b: F2 minimum in *c*
maxF1b: F1 maximum in *c*	maxF2b: F2 maximum in *b-c*
D1a: maxF1a – minF1a [F1 rising displacement]	D2a: maxF2a – minF2a [F2 falling displacement]
D1b: maxF1a – minF1b [F1 falling displacement]	D2b: maxF2b – minF2a [F2 rising displacement]
D1c: maxF1b – minF1b [F1 rising displacement]	D2c: maxF2b – minF2b [F2 falling displacement]
V1a: F1 peak rising velocity, in *a-b*	V2a: F2 peak falling velocity, in *a-b*
V1b: F1 peak falling velocity, in *a-b*	V2b: F2 peak rising velocity, in *a-b*
V1c: F1 peak rising velocity, in *b-c*	V2c: F2 peak falling velocity, in *b-c*

#### Subjects and procedure

Ten native Mandarin speakers without speech or hearing problems were recruited as subjects (5 females; Mean = 27 years, *SD* = 2.5) via the University College London Psychology Pool. The recording session for each participant lasted for around half an hour. This experiment was approved by the Committee on Research Ethics at University College London. Voice portrayal/simulation method was used to induce emotions, i.e., the participants were asked to imagine themselves in emotion-triggering scenarios when recording the sentences. This is because compared to other emotional speech induction methods (e.g., natural vocal expression), this method is more effective in obtaining relatively genuine emotional speech when experimental control is a key concern. Support for this method comes from the fact that natural emotional expression is often inherently involved with unintended portrayal and self-representation (Scherer, 2003). The recording was conducted in a sound-controlled booth. Participants were asked to record each sentence 3 times in four emotions: anger, fear, happiness, and sadness, resulting in 10 (speakers) ^*^ 4 (emotions) ^*^ 3 (tones) ^*^ 2 (segments) ^*^ 3 (repetitions) = 720 tokens.

Similar to the first experiment, a follow-up perception validation test was conducted: twenty native speakers of Mandarin (11 females, Mean = 23 years, *SD* = 2.6) were asked to rate each emotion ^*^ tone ^*^ segment token on a 1–5 scale. 1 represents not at all angry/fearful/happy/sad while 5 represents very angry/fearful/happy/sad. The top eight ranked tokens (out of 30) for each emotion ^*^ tone ^*^ segment were selected. The mean score for each emotion ^*^ tone ^*^ segment was 4.16. ProsodyPro and FormantPro scripts (Xu, 2014) running under Praat (Boersma and Weenink, 2013) was used for data analyses.

#### Results

The mean Vp/d of all measurement points are represented in Table 7. A Three-Way repeated measures ANOVA shows that among the three factors (emotion, tone, and segments), emotion is the only factor exerting a significant impact on the value of Vp/d. The interaction between emotion, tone and segments is non-significant. However, the interactions between emotion and tone and that between emotion and segments are significant (Table 8).

**Table 7:** **Mean Vp/d of the four levels of emotion (anger; fear; happiness; sadness) and the four levels of articulatory constraints (SS, small segmental distance /əu/; ST, small tonal pressure T3 + T1; LS, large segmental distance /au/; LT, large tonal pressure T2 + T2/T4 + T4)**.

	**Anger**	**Fear**	**Happiness**	**Sadness**
Mean Vp/d	37.3	41.5	43.6	24
Standard deviation	6.2	5.5	5.6	4.9
	**SS**	**ST**	**LS**	**LT**
Mean Vp/d	34	33.6	38.5	39.8
Standard deviation	5.9	4.8	5.1	5

**Table 8:** **Results of the Three-Way repeated-measures ANOVA on articulation dynamics (as reflected by Vp/d)**.

	***F***	***Df***	***P***	***η*^2^_*p*_**
Emotion	5.22	3,21	<0.01	0.25
Emotion ^*^ tone	2.39	6,42	<0.05	0.11
Emotion ^*^ segments	3.08	3,21	<0.05	0.12

*Post-hoc* Tukey tests show more details: sadness has significantly (*p* < 0.05) the lowest Vp/d value compared with the other three emotions. Happiness has the highest dynamics followed by fear and anger, but the differences between each other are non-significant.

**Figure 8:**
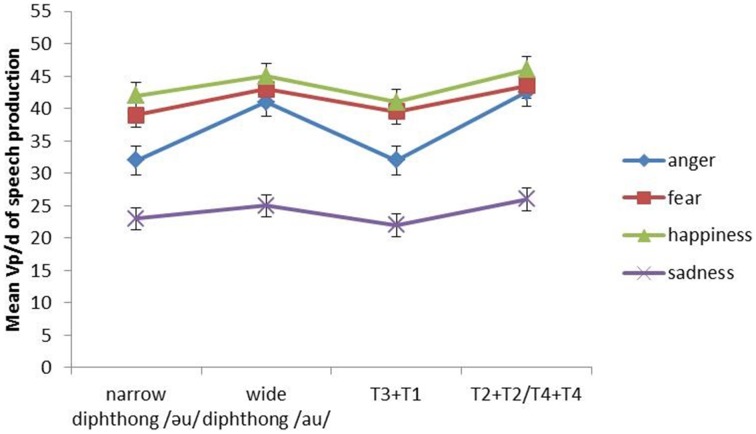
**The mean Vp/d of narrow diphthong, wide diphthong, T3 + T1 and T2 + T2/T4 + T4 in the four types of emotional speech (anger, fear, happiness, and sadness)**. Error bars represent the standard error of the mean.

The interaction between emotion and tonal pressure is significant. As shown in Figure 8, the Vp/d of all emotions is higher in tonal combinations of large articulatory constraints (i.e., T2 + T2 and T4 + T4) than the Vp/d in those of small articulatory constraints (T3 + T1). This is the most obvious in the case of anger where T2 + T2 and T4 + T4 make the Vp/d of anger become closer to that of fear and happiness. *Post-hoc* Tukey tests show that the difference between anger and fear plus that between anger and happiness are non-significant under the T2 + T2 and T4 + T4 conditions. In contrast, under the T3 + T1 condition, the differences are significant (both *p*s < 0.05). In addition, fear, happiness and sadness do not differ significantly between the two tonal conditions. Therefore, anger is more affected by tonal variation than the other three emotions.

Emotion also interacts significantly with segmental distance. Figure 8 shows Vp/d is overall higher in the wide diphthong condition than in the narrow condition. The interaction is the most obvious in anger because it is almost as high as fear and happiness with regard to Vp/d in the wide diphthong condition. *Post-hoc* Tukey tests show the difference between anger and fear plus that between anger and happiness are non-significant in the wide diphthong condition. The differences are significant (both *p*s < 0.05), however, under the narrow condition. Moreover, fear, happiness and sadness do not differ significantly between the two segmental distance conditions. Therefore, similar to above, anger is more influenced by segmental distance variation than the other three emotions.

### Comparisons between the results of the piano and speech experiment

To directly compare the results of the piano and speech experiments, a MANOVA test was conducted: the within-subjects independent variables are emotion (four levels: anger, fear, happiness, and sadness) and physical constraint (two levels: large hand span/articulatory constraints and small hand span/articulatory constraints) while the between-subjects independent variable is group (two levels: pianists and speakers). The dependent variables are Vp/d (speakers) and Vp/d (pianists). Using Pillai's trace, there is a significant difference between pianists and speakers [*F*_(8, 7)_ = 13.78, *p*<0.01]. The following univariate ANOVAs show that the group differences between pianists and speakers are significant across most conditions: anger-large [*F*_(1, 14)_ = 14.92, *p*<0.01], anger-small [*F*_(1, 14)_ = 16.23, *p*<0.01], happiness-small [*F*_(1, 14)_ = 15.61, *p*<0.01], fear-large [*F*_(1, 14)_ = 14.95, *p*<0.01], fear-small [*F*_(1, 14)_ = 18.09, *p*<0.01], sadness-large [*F*_(1, 14)_ = 15.93, *p*<0.01]. In the happiness-large and sadness-small conditions, the group difference is non-significant (speech production still has higher Vp/d than that of piano performance). The results suggest on the whole, piano performance has significantly different (i.e., lower) dynamics than speech production.

## Discussion

### Similarities between affective piano performance and speech production

The results show that firstly, anger in piano performance generates the highest dynamics irrespective of fingerings; in speech production, it is also relatively high in dynamics although it interacts with articulatory constraints (more discussions on the interaction are offered in the following section). This is in line with previous reports that anger in music performance and speech production is generally linked to fast tempo, high intensity and great energy (cf. Juslin and Sloboda, 2013). Physiologically, the high dynamics of anger can be associated with high levels of cardiovascular activities such as high heart rate (Rainville et al., 2006), fast/deep breathing (Boiten et al., 1994), increases in diastolic pressure and activated baroreceptor mechanisms (Schwartz et al., 1981). Evolutionarily, anger originates from natural and sexual selection pressure on animals (Darwin, 1872): anger induces the inclination to fight or attack whatever that threatens survival and well-being. As a result, anger is proposed to be associated with large body size projection (Morton, 1977; Xu et al., 2013a,b) to scare off enemies. Hence anger should be linked to high dynamics which can be reflected by high physical or vocal effort to show great strength and energy (Xu et al., 2013a). The results of this study support this prediction by demonstrating that greater finger force and articulatory effort are generated respectively in piano performance and speech production in the context of anger.

Secondly, happiness triggers the highest dynamics for speech production and second highest dynamics for piano performance, irrespective of fingerings or articulatory constraints. The results are in line with previous reports that in music performance, happiness is always associated with faster tempo and higher intensity (Gabrielsson, 1995; Zanon and De Poli, 2003a,b; Widmer and Goebl, 2004); happy speech is reported to have high values in many acoustic dimensions such as pitch, pitch range, intensity, high frequency energy (Scherer, 2003; Ververidis and Kotropoulos, 2006), speech rate, and formant shift (Xu et al., 2013a). Similar to anger, the physiological reason for high dynamics of happiness is often linked to increases in heart rate, blood pressure, breathing pattern (Boiten et al., 1994; Rainville et al., 2006), all of which can contribute to greater physical or vocal force in music performance or speech production. From an evolutionary perspective, happiness can be a useful strategy for attracting mates (Darwin, 1872). Therefore, it is beneficial for sound signalers to produce highly vigorous (i.e., dynamic) sounds so as to be audible to potential mates (Xu et al., 2013a). Hence, the results are also consistent with the evolutionary account.

Thirdly, fear in both piano performance and speech production produces significantly higher dynamics than sadness; particularly in speech production fear does not differ significantly from anger/happiness. This might seem somewhat unexpected given that fear in music performance is generally associated with soft playing similar to sadness (cf. Juslin and Sloboda, 2013). In terms of speech production, however, fear has already been found to show high dynamics (Xu et al., 2013a), which is consistent with the view that evolutionarily, fear can be a defensive emotion (LeDoux, 1996), evidenced from animal alarm calls as a useful antipredator defensive strategy across many species for the sake of group survival (Caro, 2005). To serve this purpose, alarm calls should be reasonably high in dynamics (i.e., vigorousness). Similarly, musical excerpts of fear could also be highly dynamic, analogous to human fearful speech or animal alarm calls.

Fourthly, sadness always generates the lowest dynamics for both piano and speech performance regardless of fingerings or articulatory constraints. This finding is in line with previous research: sad music and speech are low in intensity, F0, F0 range and duration (Juslin and Laukka, 2003; Laukka et al., 2005; Patel, 2008). This is mainly because sadness is located at the opposite end of happiness in terms of valence and arousal: it is a lowly aroused negative emotion because of its association with reduced physiological energy and arousal level, sometimes leading to affective pathology such as depression or anhedonia (cf. Huron, 2011). Evolutionarily, such low dynamics of sadness indicate a tendency for the sound signaller to beg for sympathy (Xu et al., 2013a). Hence usually low motor effort is involved in expression of sadness either through music or speech. It is worth mentioning sad speech can be split into two categories: depressed sadness and mourning sadness (Scherer, 1979), the former being characterized by low vocal energy while the latter by high vocal energy. In this study, it was the depressed sadness that was used and hence the resulting formant dynamics are low, reflecting decreased articulatory effort due to the sluggishness of articulatory muscles in sad speech (Kienast and Sendlmeier, 2000).

### Differences between affective piano performance and speech production

The results also show significant differences between the two domains. The most notable difference is that speech production on the whole has higher dynamics than piano performance across almost all conditions. This is consistent with previous studies on comparisons between speech articulatory movements and limb movements (Gentil and Tournier, 1998; Ito et al., 2004; Loucks et al., 2010). Although those studies did not investigate movements in the context of affective piano performance or speech production, the general biophysical mechanisms of fingers and speech articulators apply to this study. More specifically, it was found that compared with fingers or arms, speech articulators in general produce faster velocity (Ito et al., 2004; Loucks et al., 2010) and greater force (Gentil and Tournier, 1998; Loucks et al., 2010). The reasons probably lie in the biomechanical differences between speech articulators and fingers: compared with speech articulators, fingers are associated with more intervening factors (e.g., long tendons, joints and muscle mass between muscle fibers and skeletal joints) that prevent finger muscles from contracting as fast as speech articulatory muscles (Gentil and Tournier, 1998). It has also been reported that oral-facial muscles are associated with fast-twitch fibers and motor protein such as myosin which enable fast acceleration and rapid speech in order to meet different levels of speech demand (Williams and Warwick, 1980; Burke, 1981). Therefore, in this study the dynamics of affective speech production (as reflected by articulatory effort) and piano performance (as reflected by finger force) are different from each other due to the biomechanical distinctions.

In addition, the results also demonstrate that the interaction between emotion and physical constraints in piano performance is different from that in speech production. In piano performance (Figure 5), only fear interacts with physical constraints (i.e., fingerings); in speech production (Figure 8), only anger interacts with physical constraints (i.e., articulatory constraints). The reasons could be attributed to the differences in the extent of acoustic stability of music performance and speech production in different emotions.

Firstly, in music performance, anger, happiness, and sadness are associated with relatively consistent acoustic patterns (Juslin and Sloboda, 2013), i.e., anger and happiness are always fast and loud to convey high energy and arousal while sadness is always slow and quiet to convey low energy and arousal. Fear, in contrast, is linked to highly variable acoustic patterns especially in terms of tempo and intensity (Juslin and Madison, 1999; Madison, 2000; Juslin and Sloboda, 2013; Bernays and Traube, 2014) so as to convey the unstable psychological state under the influence of fear, e.g., scattered notes with pauses between musical phrases and sharp contrasts between intensity are often used to express fear (Madison, 2000). This could further imply there may not be a consistent pattern of finger force under the condition of fear. Hence, other factors such as fingerings are highly likely to interact with fear to generate different kinematic dynamics in piano performance.

On the other hand, fearful speech shown in this study always has high formant dynamics regardless of articulatory constraints. This is likely associated with duration. Upon close examination, the duration of fear (mean = 555.6 ms) is similarly short to that of happiness (mean = 546.6 ms) which has the highest dynamics, with non-significant differences between the two. Moreover, fear is significantly (*p* < 0.05) shorter than anger (mean = 601.1 ms) and sadness (mean = 638.2 ms). Similar findings have been reported that fear is often produced with fast speech rate that is likely to trigger vowel undershoot (i.e., an articulatory phenomenon where the canonical phonetic forms of speech sounds fail to be reached because of the articulatory impact of surrounding segments, Lindblom, 1963) and segmental reduction (Paeschke et al., 1999; Kienast and Sendlmeier, 2000). Shorter duration is highly likely to trigger great articulatory effort according to the report of studies on articulatory movement (Munhall et al., 1985; Ostry and Munhall, 1985; Edwards et al., 1991; Adams et al., 1993; Perkell et al., 2002). Therefore, the relatively stable acoustic pattern (i.e., duration) of fearful speech could make it less likely to interact with other factors such as articulatory constraints.

Secondly, this study shows that only angry speech significantly interacts with articulatory constraints: the formant dynamics are significantly higher in large articulatory constraints than those in small articulatory constraints. Again this can be linked to duration. A closer look at the data reveals that the duration of angry speech is significantly (*p* < 0.05) shorter under the condition of large articulatory constraints than the condition of small articulatory constraints. It has been reported (Cheng and Xu, 2013) that when time is short for the articulatory execution of segments with large articulatory constraints, muscles have to contract faster (i.e., with stronger articulatory effort) than when small articulatory constraints are involved in order to reach the tonal and segmental targets. This is reflected in the high formant dynamics under the condition of large articulatory constraints. In addition, the result is also consistent with the finding that anger is often more variable in duration compared with the other three emotions (happiness, fear and sadness): it can be slow because of the need to be precise and clear in articulation (Paeschke et al., 1999; Kienast and Sendlmeier, 2000) so as to project big body size to threaten away enemies (Xu et al., 2013a,b); it can also be fast in speech rate (Scherer, 2003) especially in female speakers to reflect the highly aroused and variable psychological state under the influence of anger. Hence, it is the relatively high variability in duration that makes angry speech more prone to interact with external factors such as articulatory constraints.

## Conclusion

This study compares the dynamics of piano performance (i.e., finger force) and those of speech production (i.e., articulatory effort) in four emotions: anger, happiness, fear and sadness. The results show that firstly, in both piano performance and speech production, anger and happiness generally have high dynamics while sadness has the lowest dynamics. The findings echo the theoretic argument that affective music shares a “common code” with affective speech (Juslin and Laukka, 2003). Secondly, the interaction between fear and fingerings in piano performance and the interaction between anger and articulatory constraints in speech production suggest that the more variable an emotion is in acoustic features, the more likely it is to interact in production with external factors such as fingerings or articulatory constraints in terms of dynamics. In addition, the results suggest that affective speech production on the whole has higher dynamics than affective piano performance, which may be due to the biomechanical differences between speech articulators and fingers.

Therefore, this is the first study to quantitatively demonstrate the importance of considering motor mechanisms such as dynamics (i.e., finger force and articulatory effort) together with physical constraints (i.e., fingerings and articulatory constraints) in examining the similarities and differences between affective music performance and speech production. Limitations also exist: The emotion induction method of the piano and speech experiment still needs improvement due to lack of authenticity under the laboratory condition. Moreover, more fingering strategies especially those involving black keys and more articulatory variations in speech such as monophthongs vs. diphthongs could be added to research designs for more comprehensive findings. In addition, more categories of emotion such as disgust and sarcasm could be included to make the picture more complete. In a nutshell, focusing on the motor mechanisms of affective music performance and speech production could further enhance the connection between music and speech as two fundamental capacities for humans to communicate emotion.

### Conflict of interest statement

The authors declare that the research was conducted in the absence of any commercial or financial relationships that could be construed as a potential conflict of interest.
